# Immune-related adverse events of COVID-19 vaccination in skin cancer patients receiving immune-checkpoint inhibitor treatment

**DOI:** 10.1007/s00262-021-03133-w

**Published:** 2021-12-23

**Authors:** Sophia B. Strobel, Devayani Machiraju, Katharina A. Kälber, Jessica C. Hassel

**Affiliations:** Department of Dermatology and National Center for Tumor Diseases, University Hospital Heidelberg, Im Neuenheimer Feld 460, 69120 Heidelberg, Germany

**Keywords:** COVID-19 vaccination, Cancer patients, Immune-checkpoint inhibitors, Adverse events

## Abstract

To date, few data are available regarding Adverse events (AEs) in cancer patients who are vaccinated for coronavirus disease 2019 (COVID-19) while being actively treated with Immune-checkpoint inhibitors (ICIs). We aimed to assess the safety of COVID-19 vaccines approved in Germany. Specifically, we investigated the frequency of general side effects and immune-related AEs of COVID-19 vaccination. A triage survey was used to collect the following information for patients with metastatic skin cancer: vaccine type, date of receipt of each dose of vaccine, and self-reported side effects. Clinical data were retrieved from the patients’ medical records. Of 130 patients with metastatic skin cancer, 89 patients were on immunotherapy and received COVID-19 vaccination. Of these 89 patients (median age: 64 years; 57 [64%] men), 89% had melanoma, and 71% received ICI therapy with a PD-1 antibody. Eighty-eight percent received an mRNA-based COVID-19 vaccination. The median follow-up time was 125 days after the first vaccination, and 84 days after the second. The most common observed side effects were mild to moderate pain at the injection site (40%), followed by fatigue (24%). Grade 3 irAEs were reported in eight patients, seven of whom were on nivolumab plus ipilimumab combination treatment. Of the 19 patients vaccinated within 72 h before/after ICI, five developed irAEs within 17 days (1–17 days). This small cohort study suggests that approved COVID-19 vaccinations are safe for use in cancer patients receiving ICIs. However, some precautions should be taken, especially regarding the timing of vaccination and ICI treatment.

**Supplementary Information:**

The online version contains supplementary material available at 10.1007/s00262-021-03133-w.

## Introduction

The spread of severe acute respiratory syndrome coronavirus 2 (SARS-CoV-2) has had an unprecedented deadly impact on human life. Therapeutic vaccines for coronavirus disease 2019 (COVID-19) have been developed rapidly and have proven to reduce the risk of severe illness from COVID [1].

Because cancer patients have a weakened immune system, they are at particularly high risk of infection from COVID-19; as a result, they have been prioritized under the COVID-19 vaccination strategy [2, 3]. However, concerns exist regarding potential interactions between COVID-19 vaccines and ongoing systemic cancer treatments, especially immunotherapy. Immunotherapy with Immune-checkpoint inhibitors (ICIs) restores efficient immune responses by blocking inhibitory receptors. On theoretical grounds, immunotherapy and COVID-19 vaccines could simultaneously boost the body’s systemic immune responses. It is assumed that this potential overlapping immunological enhancement of the two treatments could result in an increase in immune-related adverse events (irAEs) [4]. IrAEs among patients on ICIs are often mild to moderate, but they can also be severe, sometimes with fatal consequences [5]. So far, studies have shown no new safety issues for COVID-19 vaccines in patients with cancer generally, or in cancer patients being treated with ICIs; nonetheless, residual uncertainties remain [6–8]. In particular, the possibility that COVID-19 vaccines could trigger cytokine release syndrome during ICI treatment has been a cause for concern [9].

In this study, therefore, we investigate the safety and tolerability of COVID-19 vaccines approved in Germany, in cancer patients who are currently receiving ICI treatment.

## Methods

We conducted a retrospective study of advanced skin cancer patients actively receiving systemic treatment for cancer at the Dermato-Oncology Department of the National center for tumor diseases (NCT) at Heidelberg University Hospital. Between March 1, 2021, and July 11, 2021, a triage survey was used to collect the following information for these patients: vaccine type, date of receipt of each dose of vaccine, and self-reported side effects. Clinical data such as sex, age, tumor type, type of anti-cancer therapy, and immune-related side effects were retrieved from the patients’ medical records. Chi-square and likelihood-ratio tests were performed to compare differences between the patient groups. The analysis of the retrospective data was approved by the ethics committee of the Medical Faculty of Heidelberg University (S-454/2015).

## Results

### Patient characteristics

**Fig. 1:**
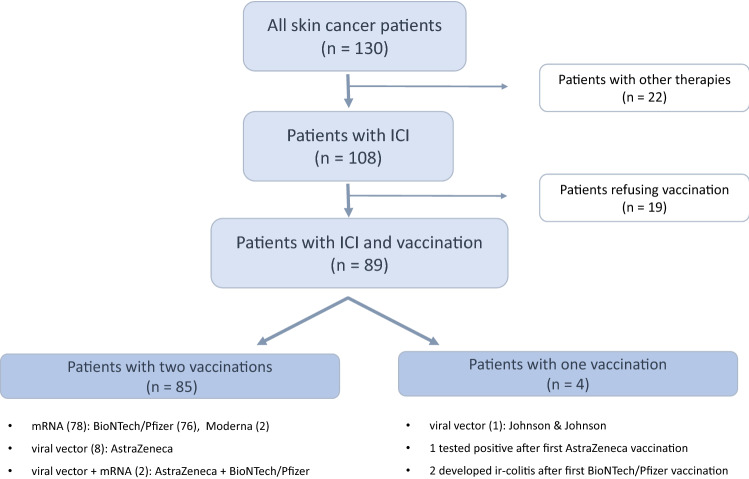
Flow Diagram of the patient cohort

In total, 130 patients with advanced skin cancer who received systemic anti-cancer treatment at the Dermato-Oncology Department of the NCT, Heidelberg, were investigated in this study (Fig. 1). Twenty-two of the 130 patients received systemic treatments other than ICI, and 19 patients refused COVID-19 vaccination. Thus, 89 patients were included in the final study population. The median age was 64 years, and 57 (64%) of the participants were men. Melanoma patients made up the largest group (89%), followed by patients with squamous cell carcinomas (7%) and Merkel cell carcinomas (4%). Seventy-one percent received a PD-1 antibody (51% pembrolizumab, 16% nivolumab, and 4% cemiplimab), 22% received a combination of CTLA4 and PD-1 antibody (ipilimumab + nivolumab), and 7% received a PD-L1 antibody (avelumab; Supplementary Table 1).

Eighty-eight percent of the patients received an mRNA vaccination (86% BioNTech and 2% Moderna), 9% received two doses of a viral vector (AstraZeneca), 2% received a combination of a viral vector and an mRNA vaccination (AstraZeneca and BioNTech), and 1% received the viral vector by Johnson and Johnson. Seventy-one patients (80%) were on ICI therapy for a median duration of 6 months (range: 0–66 months) before their first vaccination. For 10 patients (11%), immunotherapy was initiated between the first and second vaccination, and eight patients (9%) started immunotherapy a median of 3 weeks (range: 1–17 weeks) after receiving both vaccinations. Of the 89 patients, three (3%) did not receive the required second vaccination: One patient became infected with COVID-19 after the first vaccination, and two patients developed immune-related colitis. The median follow-up time was 125 days (range: 69–215) after the first vaccination, and 84 days (range: 34–188) after the second vaccination. The median interval between vaccination and the next ICI cycle was 12 days (range: 1–40 days); however, 19 patients (21%) were vaccinated within 72 h before/after an ICI cycle.

### General side effects of COVID-19 vaccination in patients receiving ICI therapy

The most common side effects of vaccination in ICI patients were mild to moderate pain at the injection site (40%) and fatigue (24%), followed by headache (10%), muscle pain (9%), and fever and chills (both 7%). Patients reported more side effects after the second vaccination, especially fatigue, headache, muscle pain, and joint pain (Supplementary Table 2). Other vaccination-related side effects reported by patients included worsening of existing pain (three patients) and worsening of known fatigue (three patients).

### irAEs after COVID-19 vaccination in patients receiving ICI therapy

Before vaccination, irAEs were observed in 17 patients (19%). These included common irAEs such as rash, thyroiditis, hypophysitis, arthritis, and colitis (Table 1). Some patients experienced more than one irAE, such as both thyroiditis and arthritis. The median time to development of an irAE while receiving ICIs was 11 weeks (range: 3–107). Rare side effects such as radiculitis, sensory neuropathy, and diabetes were documented in three patients (3%). After vaccination, irAEs were documented in 15 patients (17%). These included eight cases of patient-reported grade 3 irAEs: three cases of colitis, one of hepatitis, two of myositis, one of myocarditis, and one case of both colitis and thyroiditis. Patients who experienced irAEs after COVID-19 vaccination were on ICI therapy for a median duration of 6 weeks (range 2–118) and received the COVID-19 vaccination 6 weeks (range 0–26) before the onset of the irAE. Only one patient who previously developed a rash before vaccination also developed both colitis and thyroiditis after vaccination.

**Table 1:** Immune-related adverse events before and after vaccination listed according to the affected organ

	irAE before vaccination	CTCAE grade	irAE after vaccination	CTCAE grade
Gastrointestinal				
Colitis	1 (1%)	2	4 (4%)	3
Hepatic				
Hepatitis	0 (0%)	n/a	1 (1%)	3
Cardiovascular				
Myocarditis	0 (0%)	n/a	1 (1%)	3
Endocrinal				
Hypophysitis	3 (3%)	2 + 3	1 (1%)	2
Thyroiditis	5 (6%)	2	2 (2%)	2
Diabetes	1 (1%)	3	0 (0%)	n/a
Pancreatitis	0 (0%)	n/a	1 (1%)	2
Dermatological				
Itching	3 (3%)	2	1 (1%)	2
Rash	2 (2%)	2	1 (1%)	1
Vitiligo	2 (2%)	1	0 (0%)	n/a
Neurological				
Sensory neuropathy	1 (1%)	3	0 (0%)	n/a
Radiculitis	1 (1%)	3	0 (0%)	n/a
Musculoskeletal				
Myositis	0 (0%)	n/a	2 (2%)	3
Rheumatological				
Arthritis	3 (3%)	2	2 (2%)	2

*CTCAE*, Common Terminology Criteria for Adverse Events; *irAE*, immune related adverse event; *n/a*, not applicable

All four patients with grade 3 colitis were on combination N + I treatment. The first patient received the BioNTech/Pfizer vaccination 4 months before immunotherapy and developed colitis after the second N + I cycle. The second patient developed colitis along with thyroiditis just 3 days after the first cycle of N + I, having received the second BioNTech/Pfizer vaccination while on pembrolizumab 1 month earlier. The third patient received the first dose of the BioNTech/Pfizer vaccine 18 days before N + I and developed colitis 2 weeks after the first cycle. The fourth patient developed colitis within hours of her first dose of the BioNTech/Pfizer vaccine, 1 week after the third N + I cycle.

A patient receiving N + I was diagnosed with grade 3 hepatitis 2 weeks after the second BioNTech/Pfizer vaccine. This patient received the first vaccination between the second and third ICI cycles, and the second vaccination between the third and fourth cycles.

Furthermore, two patients developed grade 3 myositis. The first patient received the second dose of the BioNTech/Pfizer vaccine 3 weeks before the initiation of N + I. After the second cycle, an eightfold increase in creatinine kinase (CK) was observed. The second patient received the second BioNTech/Pfizer vaccination before the third cycle of N + I and developed myositis within 3 weeks, with a sevenfold increase in CK but normal heart enzymes.

The last patient with grade 3 myocarditis was the only patient who received monotherapy with pembrolizumab. The second cycle of pembrolizumab was given after the second BioNTech/Pfizer vaccination, and a 12-fold increase in troponin I and T was observed within 3 weeks.

All patients responded well to corticosteroid therapy, meaning that the dose could be tapered within 1–3 months of treatment.

### Impact of interval between immunotherapy and vaccination

The median interval between COVID-19 vaccination and the previous ICI cycle was 14 days (range: 1–40 days), and the median interval between COVID-19 vaccination and the next ICI cycle after vaccination was 11 days (range: 1–141 days). Of the 89 patients, 19 received a vaccination within 72 h before or after ICI therapy. Of these 19 patients, 10 received ICIs before vaccination, and nine received ICIs after vaccination (Table 2). Interestingly, in this patient subgroup, five patients developed an irAE within 17 days (range: 1–17 days) of treatment, with one grade 3 ir-hepatitis, three grade 2 irAEs (thyroiditis, arthritis, itching), and one grade 1 rash. Interestingly, none of those patients had irAEs before vaccination.

**Table 2:** Immune-related adverse events in patients who received vaccination within 72 h of ICI treatment

	Vaccination within 72 hours (*n* = 19)		
	irAE (*n* = 5)	No irAE (*n* = 14)	*p* value
Age			0.74
Median (range)	65 (42–81)	63 (46–83)	
Sex:			0.5
Female	1 (20%)	5 (36%)	
Male	4 (80%)	9 (64%)	
Anticancer therapy:			0.39
PD-1 antibody	2 (40%)	9 (64%)	
PD-L1 antibody	0 (0%)	1 (7%)	
CTLA4 + PD-1 antibody	3 (60%)	4 (29%)	
Type of vaccine received by vaccinated patients:			0.36
BioNTech	5 (100)	11 (79%)	
Moderna	0 (0%)	0 (0%)	
AstraZeneca	0 (0%)	2 (14%)	
AstraZeneca + BioNTech	0 (0%)	1 (7%)	
Johnson & Johnson	0 (0%)	0 (0%)	
Sequence of treatment			0.7
Vaccination -ICI	2 (40%)	7 (50%)	
ICI -vaccination	3 (60%)	7 (50%)	
irAEs before vaccination			0.06
Yes	0 (0%)	5 (36%)	
No	5 (100%)	0 (0%)	

*ICI*, Immune checkpoint inhibitors; *irAE*, Immune-related adverse event.

## Discussion

Current knowledge regarding the safety and efficacy of authorized COVID-19 vaccines in patients with cancer, and particularly in those receiving ICI treatment, is limited. It is assumed that the potential enhancement of systemic immune responses from COVID-19 vaccines and ICI treatment could increase the rate of irAEs in cancer patients who are vaccinated during immunotherapy. In this study, we found that COVID-19 vaccines were well tolerated by advanced skin cancer patients treated with ICIs: The rate of general side effects was lower than that seen in preliminary clinical data from vaccination studies [10, 11].

All general vaccination side effects were classified as grade 2 and below. They were also reversible and lasted for a maximum of 10 days. The most common general side effects after the first or second vaccination in patients receiving ICIs were fatigue and pain at the injection site, which is in line with previous findings [10]. Three ICI patients explicitly noticed worsening of fatigue after vaccination. Fatigue is the AE most commonly associated with anti-PD-1/PD-L1 antibodies, with an incidence of 16–37%, and with even higher incidences when combined with other ICI agents (21–71%) [12]. Thus, the side effects attributed to vaccination could also be the result of ICI treatment.

Regarding irAEs, eight patients in total developed grade 3 irAEs (three cases of colitis, two of myositis, one of hepatitis, one of myocarditis, and one patient with colitis and thyroiditis) after vaccination. Apart from the patient with myocarditis, all received combination N + I treatment. Interestingly, two of the four patients who developed colitis experienced it after the first N + I cycle, whereas it is usually observed after the second cycle [13]. In previous studies of N + I treatment, severe irAEs (grade 3 and above) such as diarrhea and increased liver enzymes were reported in 9–10% of patients, whereas myositis was observed in < 1% of patients [14, 15]. In our study cohort, the rate of irAEs seemed to be higher in the 20 patients who received ICI combination treatment and COVID-19 vaccination. However, because the patient numbers in this group were very small, it is not possible to draw definitive conclusions, and further studies are required to assess safety in this group.

Current empirical recommendations are to avoid COVID vaccination within 48–72 h of the administration of systemic anticancer treatments. In line with this, our study suggests that the time of COVID-19 vaccination and administration of ICIs, especially for N + I combinational treatment, may slightly influence the irAE outcome, with 26% of patients who were vaccinated within 72 h developing an irAE within 17 days [16]. Furthermore, it is interesting to note that the patients with a short interval between vaccination and ICIs made up a large proportion of the patients who experienced side effects at all after COVID-19 vaccination (33%). Thus, it is advisable to continue to follow the precautionary measure of maintaining the recommended minimum time interval between vaccination and ICI therapy.

Notably, cytokine release syndrome > grade 2 was not observed. Fever, which is the main symptom of cytokine release syndrome, was reported in 7% of patients after the first vaccination, and in 12% after the second vaccination [17]. This incidence appears to be consistent with the normal rate for this AE [10, 11]. Cytokines were not measured in these patients, however, and all patients recovered within 7 days without treatment.

In summary, vaccination during immunotherapy appears to be safe. We did not detect an increased rate of irAEs. At most, fast onset of irAEs might be observed if vaccination is administered right before the initiation of N + I. Larger and longer-term observational studies are needed for a better evaluation.

## Limitations of this study

Because 88% of the patients in our study received an mRNA vaccination, it is not possible to make a definitive statement about the safety and possible frequency of irAEs for viral vector vaccinations. Furthermore, there was no defined interval between the two treatment sequences (vaccination and ICIs). Long-term irAEs also require further investigation. More studies are highly warranted to investigate the safety and tolerability of coronavirus vaccination and immunotherapy.

## Supplementary Information

Below is the link to the electronic supplementary material.

Supplementary file1 (PDF 128 kb)

Supplementary file2 (PDF 137 kb)

## Data Availability

The datasets generated and/or analyzed during the current study are available from the corresponding author on reasonable request.
