# Supplementary material for: Immune-related adverse events of COVID-19 vaccination in skin cancer patients receiving immune-checkpoint inhibitor treatment
Source: Cancer Immunol Immunother. 2021 Dec 23;71(8):2051–6. doi: 10.1007/s00262-021-03133-w (PMC8695667; doi:10.1007/s00262-021-03133-w)
Supplement: Supplementary file 1 — Supplementary file1 (PDF 128 kb) [file 262_2021_3133_MOESM1_ESM.pdf]

Supplementary Table 1: Characteristics of all cancer patients and of the patients who received COVID-19 vaccination and ICI treatment

	All patients (n=130)	ICI + vac patients (n=89)
**General information:**		
Median age (range)	63 (21-88)	64 (31-88)
Women	51 (39%)	32 (36%)
Men	79 (61%)	57 (64%)
Median BMI (range)	26 (17-43)	26 (19-34)
Vaccinated patients	109 (84%)	89 (100%)
Vaccination not wanted	20 (15%)	n/a
No vaccination because of coronavirus infection	1 (0.8%)	n/a
**Tumor type:**		
Melanoma	113 (87%)	79 (89%)
Squamous cell carcinoma	8 (6%)	6 (7%)
Merkel cell carcinoma	4 (3%)	4 (4%)
Basal cell Carcinoma	1 (1%)	0 (0%)
Angiosarcoma	2 (1.5%)	0 (0%)
Lymphoma	2 (1.5%)	0 (0%)
**Anti-cancer therapy:**		
PD-1 antibody	79 (61%)	63 (71%)
PD-L1 antibody	6 (5%)	6 (7%)
CTLA4 + PD-1 antibody	24 (18%)	20 (22%)
CD-30 antibody	1 (1%)	n/a
Targeted therapy	6 (5%)	n/a
Chemotherapy	5 (4%)	n/a
Bispecific fusion protein	8 (6%)	n/a
Vaccination	1 (1%)	n/a
**Type of vaccine received by vaccinated patients:**		
BioNTech	94 (86%)	76 (86%)
Moderna	3 (3%)	2 (2%)
AstraZeneca	8 (7%)	8 (9%)
AstraZeneca + BioNTech	2 (2%)	2 (2%)
Johnson & Johnson	2 (2%)	1 (1%)
**Follow-up time after vaccination:**		
Median FU after first vaccination (range)	125 days (49-215)	125 days (69-215)
Median FU after second vaccination (range)	84 days (31-188)	84 days (34-188)

Abbreviations: BMI, body mass index. FU, follow-up. ICI, immune checkpoint inhibitors. n/a, not applicable. vac, vaccinated.
