# Supplementary material for: Immune-related adverse events of COVID-19 vaccination in skin cancer patients receiving immune-checkpoint inhibitor treatment
Source: Cancer Immunol Immunother. 2021 Dec 23;71(8):2051–6. doi: 10.1007/s00262-021-03133-w (PMC8695667; doi:10.1007/s00262-021-03133-w)
Supplement: Supplementary file 2 — Supplementary file2 (PDF 137 kb) [file 262_2021_3133_MOESM2_ESM.pdf]

Supplementary Table 2: General side effects reported by all metastatic skin cancer patients and by ICI patients after first and second vaccination, with duration of symptoms given in days

	All patients				ICI patients			
**Symptoms**	**1st vaccination (n=109)**	**Median duration in days (range)**	**2nd vaccination (n=102)**	**Median duration in days (range)**	**1st vaccination (n=89)**	**Median duration in days (range)**	**2nd vaccination (n=85)**	**Median duration in days (range)**
Pain at injection site	44 (40%)	1 (1-7)	38 (37%)	1 (1-7)	36 (40%)	1 (1-7)	32 (38%)	1 (1-7)
Redness at injection site	7 (6%)	2 (1-4)	4 (4%)	3 (1-7)	6 (7%)	2 (1-4)	4 (5%)	3 (1-7)
Fatigue	23 (21%)	1 (1-10)	33 (32%)	2 (1-7)	21 (24%)	1 (1-10)	32 (38%)	2 (1-7)
Feeling of weakness	5 (6%)	5 (2-10)	13 (13%)	2 (1-7)	5 (6%)	5 (2-10)	13 (15%)	2 (1-7)
Malaise	2 (2%)	2 (1-3)	10 (10%)	1.5 (1-7)	2 (2%)	2 (1-3)	9 (11%)	2 (1-7)
Vomiting	0 (0%)	n/a	3 (3%)	1	0 (0%)	n/a	3 (4%)	1
Dizziness	4 (4%)	2.5 (1-10)	5 (5%)	2 (1-5)	4 (4%)	2.5 (1-10)	5 (6%)	2 (1-5)
Headache	9 (8%)	2 (1-7)	15 (15%)	1 (1-6)	9 (10%)	2 (1-7)	15 (18%)	1 (1-6)
Muscle pain	9 (8%)	2 (1-7)	11 (11%)	1 (1-7)	8 (9%)	2 (1-7)	10 (12%)	1.5 (1-7)
Joint pain	5 (5%)	5 (2-7)	10 (10%)	1.5 (1-7)	5 (6%)	5 (2-7)	10 (12%)	2 (1-7)
Fever	6 (7%)	1.5 (1-7)	11 (11%)	1 (1-7)	6 (7%)	1.5 (1-7)	10 (12%)	1.5 (1-7)
Chills	4 (4%)	1 (1-7)	7 (7%)	1 (1-3)	6 (7%)	1.5 (1-7)	6 (7%)	1.5 (1-3)
Flu-like symptoms	3 (3%)	4 (1-7)	4 (4%)	2 (2-5)	3 (3%)	4 (1-7)	4 (5%)	2 (2-5)
Lymph node swelling	1 (1%)	7	2 (2%)	4.5 (2-7)	1 (1%)	7	1 (1%)	7
Heart racing	0 (0%)	n/a	1 (1%)	5	0 (0%)	n/a	1 (1%)	5
Rash	0 (0%)	n/a	0 (0%)	n/a	0 (0%)	n/a	0 (0%)	n/a
Seizure	0 (0%)	n/a	0 (0%)	n/a	0 (0%)	n/a	0 (0%)	n/a
Allergic reactions	0 (0%)	n/a	0 (0%)	n/a	0 (0%)	n/a	0 (0%)	n/a

Abbreviations: ICI, immune checkpoint inhibitors. n/a, not applicable.
